# Dissecting the genetic architecture of suicide attempt and repeated attempts in Korean patients with bipolar disorder using polygenic risk scores

**DOI:** 10.1186/s40345-022-00251-x

**Published:** 2022-02-03

**Authors:** Dongbin Lee, Ji Hyun Baek, Kyooseob Ha, Eun-Young Cho, Yujin Choi, So-Yung Yang, Ji Sun Kim, Yunji Cho, Hong-Hee Won, Kyung Sue Hong

**Affiliations:** 1 Department of Psychiatry, Samsung Medical Center, Sungkyunkwan University School of Medicine, 81 Irwon-ro, Gangnam-gu, Seoul, 06351 South Korea; 2 Department of Digital Health, Samsung Advanced Institute for Health Sciences and Technology (SAIHST), Sungkyunkwan University, Samsung Medical Center, Seoul, South Korea; 3 Department of Psychiatry, Seoul National University College of Medicine, Seoul, South Korea; 4 Institute of Human Behavioral Medicine, Seoul National University College of Medicine, Seoul, South Korea; 5 Samsung Biomedical Research Institute, Seoul, South Korea; 6 Department of Psychiatry, NHIS Ilsan Hospital, Goyang-si, Gyeonggi-do South Korea; 7 Department of Psychiatry, Soonchunhyang University Cheonan Hospital, Cheonan, South Korea

**Keywords:** Suicide attempt, Repeated attempts, Bipolar disorder, Polygenic risk score, Obsessive–compulsive disorder

## Abstract

**Background:**

Bipolar disorder (BD) has the greatest suicide risk among mental and physical disorders. A recent genome-wide association study (GWAS) of European ancestry (EUR) samples revealed that the genetic etiology of suicide attempt (SA) was not only polygenic but also, in part, diagnosis-specific. The authors aimed to examine whether the polygenic risk score (PRS) for SA derived from that study is associated with SA or repeated attempts in Korean patients with BD. This study also investigated the shared heritability of SA and mental disorders which showed an increased risk of SA and a high genetic correlation with BD.

**Methods:**

The study participants were 383 patients with BD. The history of SA was assessed on a lifetime basis. PRSs for reference disorders were calculated using the aforementioned GWAS data for SA and the Psychiatric Genomics Consortium data of BD, schizophrenia, major depressive disorder (MDD), and obsessive–compulsive disorder (OCD).

**Results:**

The PRS for SA was significantly associated with lifetime SA in the current subjects (Nagelkerke’s R^2^ = 2.73%, odds ratio [OR] = 1.36, *p* = 0.007). Among other PRSs, only the PRS for OCD was significantly associated with lifetime SA (Nagelkerke’s R^2^ = 2.72%, OR = 1.36, *p* = 0.007). The PRS for OCD was higher in multiple attempters than in single attempters (Nagelkerke’s R^2^ = 4.91%, OR = 1.53, *p* = 0.043).

**Conclusion:**

The PRS for SA derived from EUR data was generalized to SA in Korean patients with BD. The PRS for OCD seemed to affect repeated attempts. Genetic studies on suicide could benefit from focusing on specific psychiatric diagnoses and refined sub-phenotypes, as well as from utilizing multiple PRSs for related disorders.

**Supplementary Information:**

The online version contains supplementary material available at 10.1186/s40345-022-00251-x.

## Background

Suicide is a major global public health problem. Most suicide attempts (SAs) or completed suicides occur in persons with mental disorders (Mann et al. 2005). Among them, bipolar disorder (BD) shows the highest suicide risk, which is 10- to 30-fold greater than that of the general population (Novick et al. 2010; Pompili et al. 2013; Schaffer et al. 2015; Tondo et al. 2016). This can be explained by both a high rate of lifetime suicide attempt and a high standardized mortality ratio (SMR) for suicide in patients with BD (Novick et al. 2010; Schaffer et al. 2015). Up to half of the patients with BD attempt suicide at least once in their lifetime, and about one-third of them die by suicide (Chen and Dilsaver 1996; Gonda et al. 2012; Plans et al. 2019). Repeated suicide attempts are also most common in patients with BD among those with mental disorders, contributing to the high SMR for suicide (Jeon et al. 2010; Papadopoulou et al. 2020). However, the biological basis of suicide has been fully explored in neither patients with BD nor the general population.

The heritability estimate of suicidal behavior largely varies from 17 to 55% according to family and twin studies (Statham et al. 1998; Roy and Segal 2001; Brent and Mann 2005; Voracek and Loibl 2007), reflecting its complexity in terms of clinical manifestations (Glenn et al. 2018; Malhi et al. 2018) and genetics. Genetic mechanisms related to suicide might differ depending upon specific phenotypes such as suicidal ideation (SI) or SA (Brent and Mann 2005; Mann et al. 2005; Mullins et al. 2014), and on underlying mental disorders. Also, multiple attempters showed demographic and clinical characteristics different from those of single attempters (Jeon et al. 2010; Fedyszyn et al. 2016; Arici et al. 2018; Icick et al. 2019; Papadopoulou et al. 2020). In BD, previous studies reported multiple psychiatric comorbidities and substance abuse as clinical factors associated with repeated attempts (Arici et al. 2018; Icick et al. 2019). However, most genetic studies on SA have used a single phenotype defined as “at least one SA in a lifetime” (Mullins et al. 2014; Mullins et al. 2019; Erlangsen et al. 2020; Ruderfer et al. 2020). Applying more specific sub-phenotypes in exploring the complex genetic architecture of SA is needed.

Two recent large-scale genetic studies addressed the issue of whether the genetic susceptibility for suicide was shared in common or distinct between various psychiatric illnesses. Erlangsen et al. (2020) calculated the single nucleotide polymorphism (SNP) heritability (*h*^2^) of SA in different diagnostic groups including major depressive disorder (MDD), schizophrenia (SCZ), anorexia, autism, attention-deficit/hyperactivity disorder (ADHD) and BD. It varied greatly across diagnoses, showing the highest value in BD. Mullins et al. (2019) performed the largest genome-wide association study (GWAS) for SA in European ancestry (EUR) patients with MDD, SCZ, or BD. In this study, a significant locus in the discovery cohort with mixed diagnosis had the strongest effect in the BD subgroup, and its association was not replicated in another cohort with predominantly MDD diagnosis. Also, the polygenic risk score (PRS) for SA calculated from one disorder subgroup was not associated with SA in another disorder subgroup. This GWAS result for SA has not yet been utilized as reference data to calculate PRS for SA except in the study itself. In addition, most genetic studies of SA have been performed in EUR samples, with relatively few in other populations (Bondy et al. 2006; Galfalvy et al. 2009; Rawat et al. 2019; Gupta et al. 2020; Rao et al. 2020).

In exploring complex phenotypes like suicide, investigating the shared heritability with other psychiatric traits is a useful way to identify the genetic architecture. Given that SA is prevalent in several mental disorders, and that it co-aggregates with those mental disorders within families, overlapping genetic underpinning between SA and mental disorders are expected (Ballard et al. 2019; Too et al. 2019). A previous study applied the linkage disequilibrium score regression method using GWAS results for SA and other psychiatric traits to investigate the genetic correlation between them (Ruderfer et al. 2020). This study showed significant but incomplete correlation of SA with insomnia and several psychiatric disorders. PRS analysis is another method used to investigate shared heritability between traits by testing if the PRS for one trait shows an association with another directly assessed trait in an independent sample. PRS also provides individual-level risk estimates, which could potentially be utilized in various subsequent analyses including prediction modeling (Fullerton and Nurnberger 2019; Ikeda et al. 2021). To investigate the shared heritability of SA in BD and other psychiatric traits, GWAS results of mental disorders with stronger genetic correlations with BD and higher SA risk could be considered as high priority reference data. According to a recent report by cross-disorder group in Psychiatric Genomic Consortium (PGC) (Cross-Disorder Group of the Psychiatric Genomics Consortium 2019), SCZ, MDD, and obsessive–compulsive disorder (OCD), in that order, showed the highest genetic correlation with BD. It is also known that patients with these disorders are at high risk of SA (Too et al. 2019; Pellegrini et al. 2020).

This study aimed to explore the genetic basis of SA in patients with BD using polygenic risk analysis. Specifically, we examined the association between the PRS for SA calculated from the GWAS result of EUR patients with BD and lifetime SA and repeated attempts in Korean BD patients. In addition, the shared heritability of SA and four mental disorders, i.e., BD, SCZ, MDD, and OCD was investigated using PRS analyses.

## Methods

### Study participants

We recruited patients with bipolar I disorder (BD-I) or bipolar II disorder (BD-II) at Samsung Medical Center (SMC) and Seoul National University Bundang Hospital (SNUBH). The participant diagnoses and detailed comorbid symptoms were confirmed using DSM-IV-TR criteria adopted in the Korean version of the Diagnostic Interview for Genetic Studies (DIGS) (Joo et al. 2004) in the SMC, and the Korean version of Mini-International Neuropsychiatric Interview (MINI) (Yoo et al. 2006) in the SNUBH. The detailed evaluation process and the evaluated variables were described elsewhere (Baek et al. 2019). Written informed consent was obtained from all participants. This study was approved by the Institutional Review Boards at SMC and SNUBH.

### Assessment of suicidality

We assessed the lifetime SA and number of attempts using items from the DIGS, the MINI, and/or the suicide module of the Korean version of the Composite International Diagnostic Interview (CIDI) (Cho et al. 2002). Details on the specific items and the number of patients assessed using each tool are shown in Additional file 1: Tables S1 and S2.

### Genotyping

We used the Korea Biobank Array (Moon et al. 2019) for genotyping DNA samples. This array used an Axiom™ KORV1.0-96 Array (Affymetrix, Santa Clara, CA, USA) and was designed by the Center for Genome Science at the Korea National Institute of Health. The array was optimized for the Korean population, comprising > 833,000 markers including common and rare-frequency variants, and functional variants estimated from the sequencing data of > 2500 Koreans. Quality control (QC) was performed according to the Korea Biobank Array protocol (http://www.koreanchip.org). The quality control parameters for excluding study samples and variants were as follows: variants with variant call rate < 0.99, Hardy–Weinberg equilibrium *p* < 10^−6^, minor allele frequency (MAF) < 0.01, or duplicated SNPs, and samples with first or second degree relatedness, sample call rate < 0.95, excessive heterozygosity, sex discrepancy, or outliers in the principal component analysis. Genetic relatedness was inferred using KING (Manichaikul et al. 2010). After sample QC followed by variant QC, phasing and imputation were performed with Eagle v2.4 and Minimac 4 using the Haplotype Reference Consortium reference panel (Howie et al. 2012; Loh et al. 2016; McCarthy et al. 2016). Variants with imputation quality R^2^ < 0.8 or MAF < 0.01 were removed. Finally, 5,483,856 SNPs were used for data analysis. In later multivariate analysis, multidimensional scaling plots were reexamined conservatively to remove outlier samples. The principal components were calculated using PLINK version 1.9 (Purcell et al. 2007) for use as population stratification covariates (Additional file 1: Fig. S1).

### GWAS summary statistics

To derive PRS for SA, we used the results from only the BD samples in the GWAS by Mullins et al. (2019). For exploratory purposes, we additionally calculated PRS for SA from the SCZ samples, or from the only MDD samples of the same GWAS. We also used the most recent GWAS summary statistics from the PGC for BD (Stahl et al. 2019), SCZ (Lam et al. 2019), MDD (Howard et al. 2019), and OCD (International Obsessive Compulsive Disorder Foundation Genetics Collaborative (IOCDF-GC) and OCD Collaborative Genetics Association Studies (OCGAS) 2018). Only the GWAS for SCZ included Asian samples. Details including sample size, population, and SNP heritability of the studies are summarized in Additional file 1: Table S3.

### Data analysis

We used PRSice-2 to calculate PRSs and followed the standard protocol unless otherwise mentioned (Choi and O'Reilly 2019). We calculated the PRSs using ten different p-value thresholds for each GWAS summary statistic, and the PRSs were standardized to have a mean of zero and a standard deviation (SD) of one. For lifetime SA as the dependent variable (DV), we performed univariate logistic regression with each calculated PRS as the independent variable (IV). Nagelkerke’s pseudo R^2^ was used to measure model performance to select the best-fit model. We then performed multivariate logistic regression adjusted for age, sex, diagnosis (BD-I vs. BD-II), study center and the first two principal components.

We also conducted univariate linear regression to explore the relationship between the number of attempts (including zeros from non-attempters) and PRSs and to select the best-fit model using R^2^. Then the PRSs from the best-fit model between single attempters and multiple attempters (number of attempts ≥ 2) were compared using logistic regression. We then conducted multivariate logistic regression using all five PRSs as the IVs to account for known shared genetic architecture between the five traits.

We considered the difference between BD-I and BD-II in genetic architecture and the manifestation of suicidal behavior. We repeated univariate logistic regression analyses in the subgroups by diagnosis using each PRS as the IV and lifetime SA as the DV.

We additionally compare the rate of lifetime SA between patients with and without comorbid OCD using logistic regression.

A *p* value of less than 0.05 was considered statistically significant. All statistical analyses were performed using R version 4.02 (R Development Core Team 2010).

## Results

### Basic characteristics and suicidality of study participants

Table 1 summarizes the characteristics of the 383 patients with bipolar disorder. The mean age (SD) of the participants was 34.9 years (10.9). The proportion of females and patients with BD-I were 65%, and 59%, respectively. A history of lifetime SA was observed in 121 (31.6%) patients. Suicide attempters were more common in those with BD-II (41.1%) than in those with BD-I (24.9%). Except for the diagnosis, the basic characteristics did not differ between the attempters and non-attempters. Additional file 1: Fig. S2 illustrates the distribution of the number of attempts (total N = 378). Among 121 attempters, 64 (16.9%) were single attempters, 52 (13.8%) were multiple attempters, and five had no data on the number of attempts.

**Table 1:** Basic characteristics of the study subjects and comparison between suicide attempters (N = 121) vs. non-attempters (N = 262)

	Suicide attempters^a^ (N = 121)		Non-attempters (N = 262)		Total (*N* = 383)		*P*^b^
	Mean	SD	Mean	SD	Mean	SD	
Age	33.3	10.4	35.6	11	34.9	11	0.065

	N	%	N	%	N	%	*P*^b^
Sex, female	84	69.4	165	63	249	65	0.265
Diagnosis, bipolar II disorder	65	53.7	93	35.5	158	41	0.001
Study center, SNUBH	60	49.6	111	42.4	171	45	0.226

^a^Suicide attempters include single attempters (N = 64) and multiple attempters (N = 52)

^b^Difference was tested between suicide attempters and non-attempters using the t-test or chi-squared test

### Association between PRS for SA (PRS-SA) and lifetime SA

**Fig. 1:**
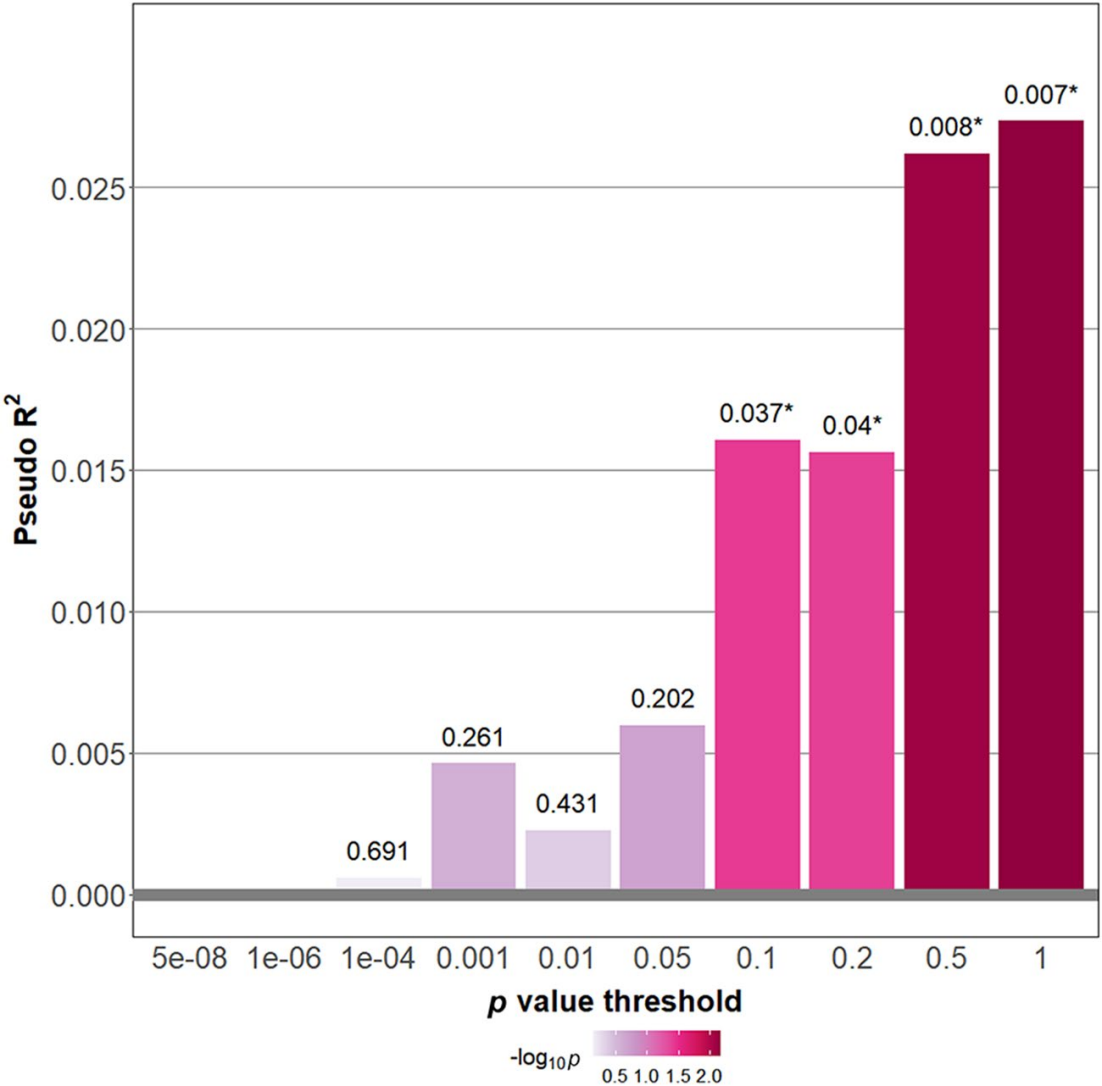
Association between polygenic risk scores for suicide attempt and lifetime suicide attempt. Logistic regression was performed with the PRS for suicide attempt as the independent variable and suicide attempters (N = 121) vs. nonattempters (N = 262) as the dependent variable. The x-axis illustrates the p-value thresholds used to filter the variants from GWAS for suicidal attempt. The y-axis illustrates Nagelkerke’s pseudo R^2^. The *p* values for the associations are shown above each bar. *Asterisk indicates nominal significance (*p* < 0.05). *PRS* polygenic risk score, *GWAS* genome-wide association study

The PRS for SA (PRS-SA) showed a significant association with lifetime SA (Fig. 1). One SD increase in the best-fit PRS was associated with a 36% increase in the odds ratio (OR) of attempters vs. non-attempters (best-fit *p* value threshold = 1, Nagelkerke’s R^2^ = 2.73%, OR = 1.36, 95% CI = 1.09–1.70, *p* = 0.007). The association remained significant after adjusting for age, sex, diagnosis, study center, and the first two principal components (PRS-SA, OR = 1.32, 95% CI = 1.05–1.66, *p* = 0.018) (Table 2). Compared with these results, the PRSs for SA calculated from only SCZ samples, or from only MDD samples did not show significant association with lifetime SA in our BD samples (Additional file 1: Fig. S6). Also, the Nagelkerke’s R^2^ values were relatively lower.

**Table 2:** Multivariate logistic regression analysis with lifetime suicide attempt as the dependent variable

	Odds ratio	95% CI	*P*
PRS for suicide attempt as the independent variable			
PRS for suicide attempt	1.32	1.05–1.66	0.018
Age	0.97	0.95–1.00	0.026
Sex, female	1.27	0.78–2.07	0.334
Diagnosis, bipolar II disorder	1.96	1.22–3.16	0.006
Study center, SNUBH	1.26	0.77–2.07	0.356
1st principal component^a^	0.29	0.00–24.07	0.587
2nd principal component^a^	0.80	0.01–66.14	0.920
PRS for obsessive–compulsive disorder as the independent variable			
PRS for obsessive–compulsive disorder	1.32	1.05–1.66	0.017
Age	0.98	0.95–1.00	0.028
Sex, female	1.29	0.79–2.09	0.312
Diagnosis, bipolar II disorder	1.99	1.24–3.21	0.005
Study center, SNUBH	1.31	0.80–2.15	0.285
1st principal component^a^	0.18	0.00–15.05	0.448
2nd principal component^a^	0.95	0.01–78.63	0.982

*PRS* polygenic risk score, *SNUBH* Seoul National University Bundang Hospital

^a^Five outlier samples were removed after visual inspection of the multidimensional scaling plots, then the principal components were recalculated

### Association between PRSs for four psychiatric disorders and lifetime SA

**Fig. 2:**
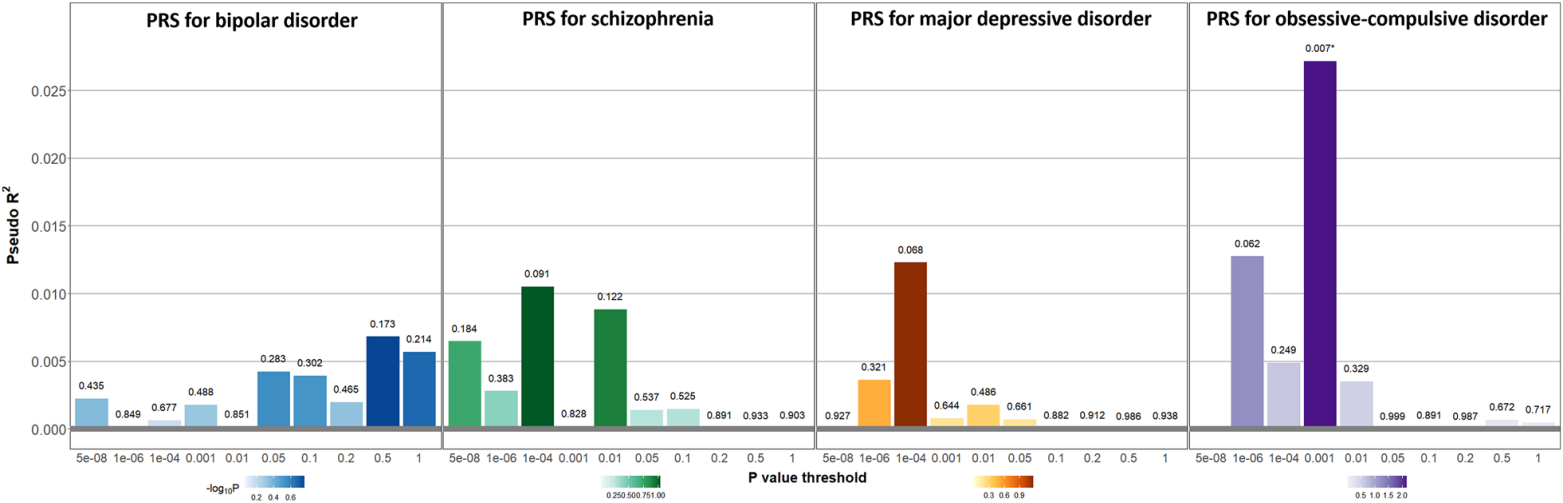
Association between polygenic risk scores for four mental disorders and lifetime suicide attempt. Logistic regression was performed with each PRS as the independent variable and suicide attempters (N = 121) vs. nonattempters (N = 262) as the dependent variable. The x-axis illustrates the *P*-value thresholds used to filter the variants from each GWAS result. The y-axis illustrates Nagelkerke’s pseudo R^2^. The *P* values for the associations are shown above each bar. *Asterisk indicates nominal significance (*p* < 0.05). *PRS* polygenic risk score, *GWAS* genome-wide association study

Neither the PRS for BD (PRS-BD), PRS for SCZ (PRS-SCZ), nor PRS for MDD (PRS-MDD) showed a significant association with lifetime SA (PRS-BD, best-fit *P*-value threshold = 0.5, Nagelkerke’s R^2^ = 0.68%, OR = 1.16, 95% CI = 0.94–1.44, *p* = 0.173; PRS-SCZ, best-fit *p* value threshold = 1 × 10^–4^, Nagelkerke’s R^2^ = 1.05%, OR = 0.83, 95% CI = 0.66–1.03, *p* = 0.091; PRS-MDD, best-fit *p* value threshold = 1 × 10^–4^, Nagelkerke’s R^2^ = 1.23%, OR = 1.23, 95% CI = 0.99–1.53, *p* = 0.068). The PRS for OCD (PRS-OCD) showed a significant association with lifetime SA (Fig. 2). One SD increase in the best-fit PRS was associated with a 36% increase in the OR of attempters vs. non-attempters (best-fit *p* value threshold = 0.001, Nagelkerke’s R^2^ = 2.72%, OR = 1.36, 95% CI = 1.09–1.69, *p* = 0.007). The association remained significant after adjusting for age, sex, diagnosis, study center, and the first two principal components (PRS-OCD, OR = 1.32, 95% CI = 1.05–1.66, *p* = 0.017) (Table 2).

### Multivariate model for lifetime SA using five PRSs

We additionally put all five PRSs into a multivariate model (Nagelkerke’s R^2^ = 8.07%). The PRS-SA and PRS-OCD still showed significant associations with lifetime SA (Table 3). The multivariate model including five PRSs, age, sex, and diagnosis showed better performance, compared to null model (including age, sex, and diagnosis) across multiple measures (Additional file 1: Fig. S3).

**Table 3:** Multivariate logistic regression analysis using five polygenic risk scores

	Odds ratio	95% CI	*P*
Multivariate model using five PRSs			
PRS for suicide attempt	1.36	1.08–1.72	0.009
PRS for bipolar disorder	1.18	0.94–1.47	0.146
PRS for schizophrenia	0.80	0.64–1.00	0.052
PRS for major depressive disorder	1.21	0.97–1.52	0.096
PRS for obsessive–compulsive disorder	1.33	1.06–1.66	0.015
Multivariate model using five PRSs with additional adjustments			
PRS for suicide attempt	1.32	1.04–1.67	0.021
PRS for bipolar disorder	1.21	0.96–1.52	0.106
PRS for schizophrenia	0.82	0.64–1.04	0.097
PRS for major depressive disorder	1.14	0.90–1.44	0.267
PRS for obsessive–compulsive disorder	1.29	1.02–1.63	0.031
Age	0.98	0.95–1.00	0.035
Sex, female	1.29	0.79–2.12	0.315
Diagnosis, bipolar II disorder	1.83	1.12–2.99	0.015
Study center, SNUBH	1.26	0.76–2.08	0.374
1st principal component^a^	0.35	0.00–32.84	0.651
2nd principal component^a^	0.86	0.01–79.46	0.947

*PRS* polygenic risk score, *SNUBH* Seoul National University Bundang Hospital

^a^Five outlier samples were removed after visual inspection of the multidimensional scaling plots, then the principal components were recalculated

### Single attempters vs. multiple attempters

Using the number of attempts as the phenotype, the best-fit PRS-SA was derived from the model using *p* value threshold of 0.5 (R^2^ = 1.68%, *p* = 0.012), while the PRS-SA using a *p* value threshold of 1 showed a comparable result (Additional file 1: Fig. S4a, b). When compared between multiple attempters and single attempters in logistic regression analysis, the chosen best-fit PRS-SA was not significantly different (*p* = 0.263) (Additional file 1: Fig. S4c, d).

**Fig. 3:**
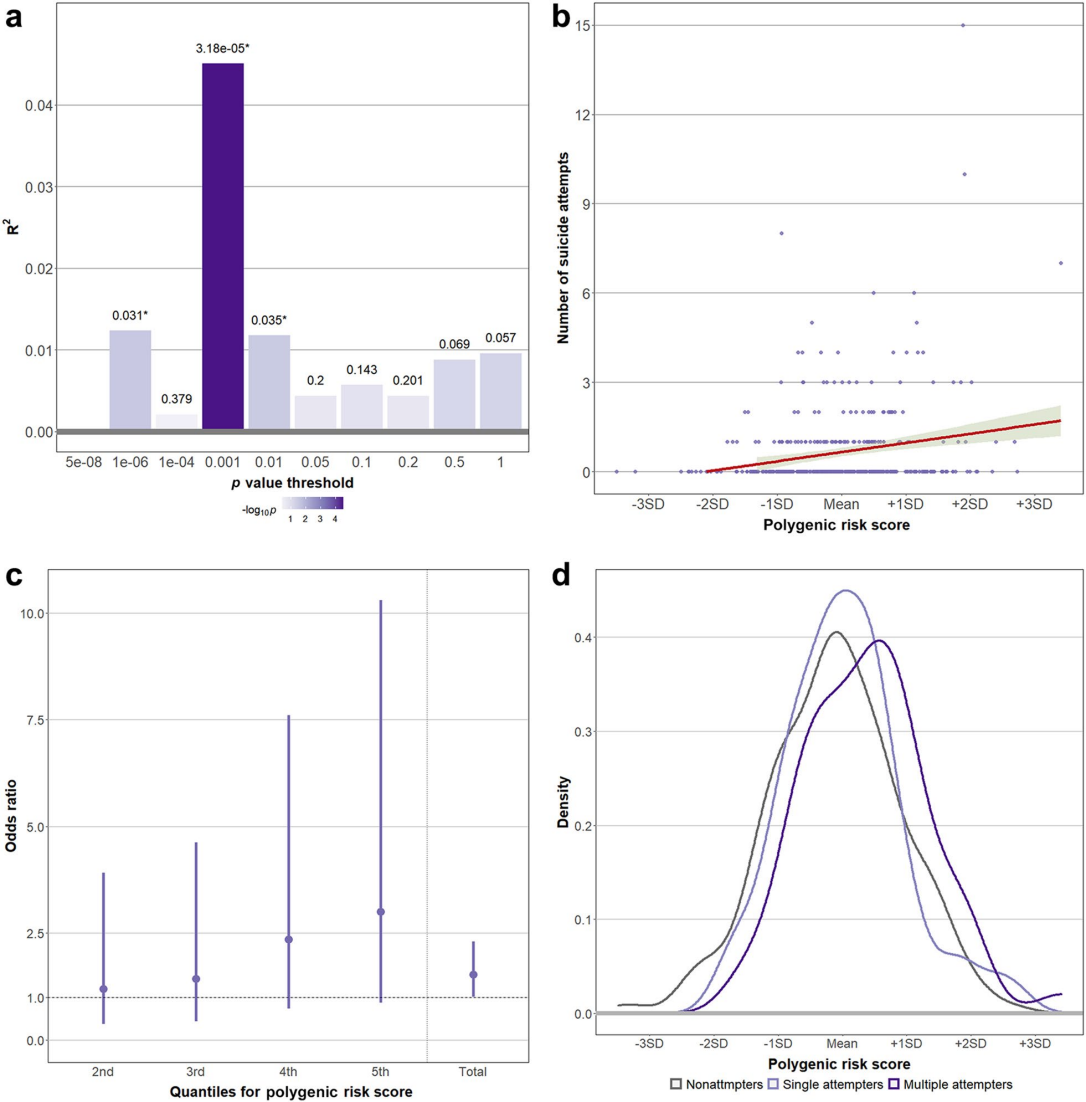
Higher polygenic risk scores for obsessive–compulsive disorder in multiple attempters (N = 64) compared to single attempters (N = 52). **A** Linear regression was performed with the PRS for OCD as the independent variable and the number of attempts as the dependent variable. The x-axis illustrates the *p* value thresholds used to filter the variants from the GWAS for OCD. The y-axis illustrates R^2^, which represents the proportion of the variance explained. The p-values for the associations are shown above each bar. *Asterisk indicates nominal significance (*p* < 0.05). Model using a *p* value threshold of 0.001 was selected as the best-fit model. **B** Scatter plot illustrates the relationship between PRS for OCD and the number of attempts. The red line and green shading illustrate the best-fit linear regression model and 95% confidence intervals, respectively. The x-axis illustrates the PRS calculated using the best-fit model. Since the PRS was standardized, x-axis ticks were marked with the mean and standard deviation. **C** Quantile plot illustrates the pattern of the ORs for multiple attempters vs. single attempters across the quantiles of the PRS for OCD. The x-axis illustrates the last four quantiles of all five quantiles of the PRS for OCD. The y-axis illustrates the OR for multiple attempters (N = 64) vs single attempters (N = 52) in each quantile compared to the first quantile. The OR from the total sample was also shown after the dotted vertical line. The points and lines illustrate the ORs and 95% confidence intervals, respectively. **D** Density plot illustrates the distribution of the PRS for OCD across non-attempters (N = 262), single attempters (N = 64), multiple attempters (N = 52), and lifetime attempters (single or multiple) (N = 121). The PRS for OCD was significantly different between single attempters vs multiple attempters, but not between non-attempters vs single attempters. *PRS* polygenic risk score, *OCD* obsessive–compulsive disorder, *GWAS* genome-wide association study, *OR* odds ratio

The PRS-OCD showed a significant association between the number of attempts in the model using a *p* value threshold of 0.001. The best-fit PRS explained 4.50% of the variance (R^2^ = 4.50%, *p* = 3.18 × 10^–5^) (Fig. 3a, b). When compared between multiple attempters and single attempters, the chosen best-fit PRS-OCD was significantly higher in the multiple attempters. One SD increase in the best-fit PRS-OCD was associated with a 53% increase in the OR for multiple attempters vs. single attempters (Nagelkerke’s R^2^ = 4.91%, OR = 1.53, 95% CI = 1.01–2.30, *p* = 0.043) (Fig. 3d). The quantile plot in Fig. 3c depicts the trend in the associations across five quantiles for PRS-OCD. In the higher quantiles compared with the first quantile, the OR for multiple attempters vs. single attempters was higher, although the association in each quantile did not reach statistical significance (quantile 2, OR = 1.20, 95% CI = 0.37–3.92; quantile 3, OR = 1.43, 95% CI = 0.44–4.62; quantile 4, OR = 2.36, 95% CI = 0.73–7.60; quantile 5, OR = 3.00, 95% CI = 0.87–10.30). In contrast, the PRS-OCD did not significantly differ between non-attempters vs. single attempters in logistic regression analysis (*p* = 0.325) (Fig. 3d).

### Subgroup analysis by diagnosis

**Fig. 4:**
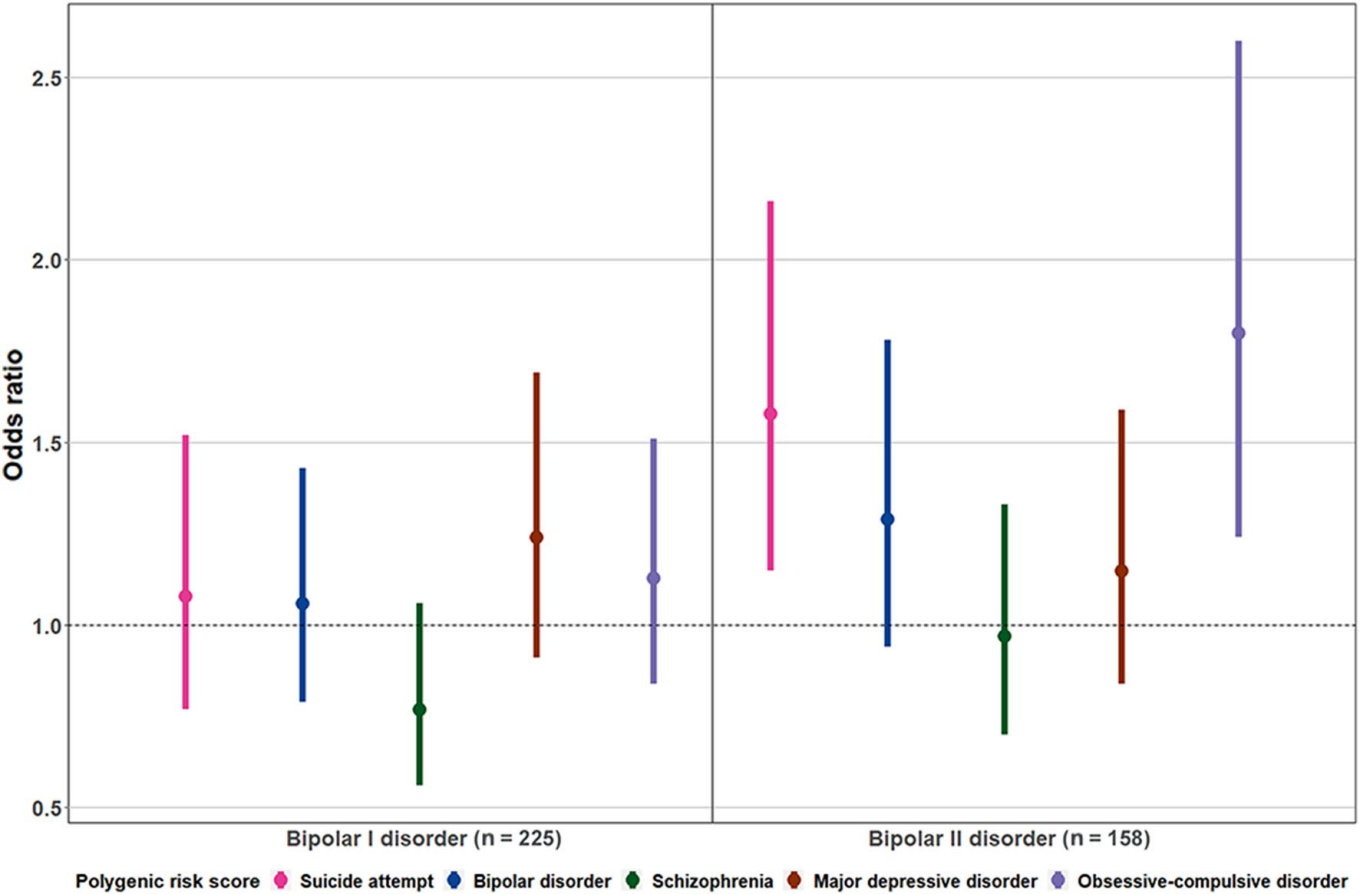
Association between five polygenic risk scores and lifetime suicide attempt in the subgroups by diagnosis. Logistic regression was performed with each PRS as the independent variable and suicide attempters vs non-attempters as the dependent variable in the subgroups by diagnosis (bipolar I disorder attempters, N = 56 and non-attempters, N = 169; bipolar II disorder attempters, N = 65 and non-attempters, N = 93). The points and lines illustrate the odds ratios and 95% confidence intervals, respectively. The PRS for suicide attempt and the PRS for obsessive–compulsive disorder showed significant associations only in the bipolar II disorder subgroup

The PRS-SA and PRS-OCD, which were significantly associated with lifetime SA in the total sample, showed significant associations with lifetime SA in BD-II subgroup (PRS-SA, OR = 1.58, 95% CI = 1.15–2.16; PRS-OCD, OR = 1.80, 95% CI = 1.24–2.60), but not in BD-I subgroup (PRS-SA, OR = 1.08, 95% CI = 0.77–1.52; PRS-OCD, OR = 1.13, 95% CI = 0.84–1.51) (Fig. 4).

### Association between comorbid OCD and lifetime SA

Among 284 patients with available clinical data, 39 (13.7%) had comorbid OCD. Comorbid OCD was not significantly associated with lifetime SA (OR = 0.74, 95% CI = 0.35–1.56, p = 0.428) or repeated attempts (OR = 0.48, 95% CI = 0.12–2.00, p = 0.316).

## Discussion

This study aimed to investigate the genetic architecture of SA in Korean patients with BD using polygenic risk scores. We applied the PRS for SA derived from the GWAS result of EUR patients with BD as well as those for four mental disorders (BD, SCZ, MDD, and OCD) based on PGC data to the current phenotype of SA and repeated attempts. The PRS-SA calculated from the EUR patients was associated with lifetime SA in the current subjects with BD. The PRS-OCD was also associated with SA, and the association was specifically significant with repeated attempts.

Even though the predictive performance of PRS usually decreases when applied to different ethnic groups (Duncan et al. 2019; Martin et al. 2019), we obtained a significant result, which indicated a 1.36-fold higher risk for suicide attempt per one SD for the PRS-SA. This significant finding might have benefited from the homogeneity of the underlying diagnosis, i.e., BD in both the discovery (Mullins et al. 2019) and target (the current) data. Owing to this specificity, however, the current result cannot be directly compared to previous findings. In most previous GWAS and PRS studies for SA, the subjects were mixed populations in terms of psychiatric diagnoses (Mullins et al. 2019; Erlangsen et al. 2020; Ruderfer et al. 2020). According to a previous study of Mullins et al. (2019), the PRS for SA calculated in one disorder was not significantly associated with SA in another disorder. Apart from that, only a few GWASs for suicide-related phenotypes have been performed in East Asian populations (Otsuka et al. 2019; Rao et al. 2020). A Japanese study (Otsuka et al. 2019) reported that polygenic risk for complete suicide calculated from a GWAS was associated with complete suicide in another cohort, showing R^2^ similar to that of our study. In that study, both discovery and target samples were homogenous Japanese population, but the psychiatric diagnoses of the subjects were not indicated. A recent Chinese study performed a GWAS of SA in patients with MDD (Rao et al. 2020). That study did not identify any SNP reaching genome-wide significance, and did not include a PRS analysis.

In the current study, the association between PRS-OCD and lifetime SA particularly reflected an association with repeated attempts (Nagelkerke’s R^2^ = 4.91% and OR = 1.53). The effect size seemed notable compared to that of previous studies on SA using PRSs for other phenotypes, e.g., MDD, schizophrenia, anxiety disorder, anhedonia, and low educational attainment (Mullins et al. 2019; Coombes et al. 2020; Lopes et al. 2020). Unfortunately, no prior study has examined the association between PRS-OCD and SA, or repeated attempts. According to a recent systematic review (Amerio 2019), whether comorbid OCD in BD was associated with an increased risk for SA at the clinical level has also yet to be confirmed. A recent study suggested comorbid OCD in patients with BD increased the risk for SA using violent methods rather than the risk for lifetime SA (Di Salvo et al. 2020). When we investigated the association between SA and comorbid OCD at the clinical level, comorbid OCD was not significantly associated with SA or repeated attempts. In addition, even though the PRS-OCD was significantly associated with both lifetime SA and comorbid OCD in the current subjects, models showing the strongest association were different between the two phenotypes, i.e., models using p-value threshold of 0.001 (Fig. 2) and 1 × 10^–6^ (Additional file 1: Fig. S5) respectively. Thus, the current data could not clarify whether the association between PRS-OCD and SA is mediated by the clinical manifestation of OCD.

Considering that obsessive–compulsive symptoms are defined as the “repetition of thought and/or behavior”, a genetic liability to OCD might affect the repetition or disinhibition of certain impulses or behaviors. According to a recent epidemiological study reporting the familial co-aggregation of OCD and suicidal behavior (Sidorchuk et al. 2021), not only OCD patients but also their unaffected relatives showed increased risks for suicide attempt or complete suicide. This implies that some traits other than overt obsessive–compulsive symptoms might mediate the association between the PRS-OCD and SA or repetition. Neurocognitive deficits could be a potential mediator given that individuals with OCD and those with suicidal behavior showed similar patterns of impairment in executive function such as response inhibition, cognitive flexibility, and decision-making (Chamberlain et al. 2007; Menzies et al. 2007; Cavedini et al. 2010; Rajender et al. 2011; Bredemeier and Miller 2015; Zhang et al. 2015; Ozcan et al. 2016; Harfmann et al. 2019). Moreover, in a recent study, PRS-OCD was associated with the personality trait of harm-avoidance (Bey et al. 2020), which was suggested as a risk factor for SA in patients with BD (Engström et al. 2004).

Recent large-scale studies (Mullins et al. 2019; Coombes et al. 2020) supported the positive association between the PRS-MDD and SA in BD samples. In our study, the trend in association did not reach statistical significance (OR = 1.23, 95% CI = 0.99–1.53). Similarly, the negative association between the PRS-SCZ and SA, which was previously found in mixed sample of BD, MDD, and SCZ (Mullins et al. 2019) did not reach statistical significance in our BD samples (OR = 0.83, 95% CI = 0.66–1.03). The present study did not show a significant association between PRS-BD and lifetime SA, which is corroborating a previous finding (Mullins et al. 2019).

Subgroup analysis by diagnosis showed different patterns of polygenic risks between BD-I and BD-II. The significant association between the PRS-SA and PRS-OCD and lifetime SA was observed only in BD-II subgroup despite the smaller sample size. In our subjects, the rate of lifetime SA was significantly higher in BD-II than in BD-I (Additional file 1: Table S4). This finding was inconsistent with those in a previous review and meta-analysis, which suggested no significant differences in lifetime SA between patients with BD-I and BD-II. However, other studies suggested that the SA patterns might be different between the two groups; several studies reported that patients with BD-II used more lethal methods compared to BD-I (Vieta et al. 1997; Tondo et al. 2007). Aaltonen et al. (2016) also reported that repeated attempts were more common in patients with BD-II. Considering the current results and differences in the genetic architecture between BD-I and BD-II reported previously (Ruderfer et al. 2014; Allardyce et al. 2018; Bipolar Disoder and Schizophrenia Working Group of the Psychiatric Genomics Consortium 2018; Stahl et al. 2019; Gordovez and McMahon 2020), genetic studies for SA need to be separately performed in patients with BD-I or BD-II using larger samples.

Since suicide is the worst outcome of mental disorders, the prediction of SA is critical in clinical practice. Traditionally, suicide risk evaluation has largely been dependent upon proximal risk factors such as depressive mood or suicidal ideation, which can be observed during the crisis state (Malhi et al. 2018). In contrast, polygenic risks can be objectively measured in the earliest phase of illness. Individual risk estimation using genomic data would improve the performance of current suicide risk evaluation. However, the PRS profiles in the present study and other genomic studies (Mullins et al. 2019; Coombes et al. 2020; Lopes et al. 2020; Ruderfer et al. 2020) explained only a small portion of the variance in SA. Therefore, to be used as a prediction model of SA, the explanatory power needs to be increased by refining suicide-related phenotypes and investigating more homogeneous populations in terms of psychiatric illnesses. In addition, the relationship between genetic factors and known clinical factors must be studied, so that they could be used together to generate a prediction model.

Several limitations must be considered when interpreting our results. First, due to the limited sample size, the data were underpowered to detect associations with relatively small effects. To validate the current data and identify more associations adopting the current design, large-scale genomic and clinical data in patients with BD and other psychiatric disorders should be accumulated. Second, we used the reference data from other ethnic groups, which could decrease the explanatory power of PRS. This may account for our result that the previously reported associations between the PRS-MDD and PRS-SCZ and SA were not replicated. Third, we did not evaluate the diverse clinical correlates of SA, which might mediate the associations observed in our study. In particular, clinical symptoms, medication effects, and the detailed characteristics of SA such as the degree of suicidal intent and lethality of the method were not investigated. Fourth, the assessment of SA was retrospective. However, a history of SA was evaluated based on a comprehensive information gathering process described elsewhere (Baek et al. 2019). In addition, even though the subjects were recruited from two hospitals using different structured interviews (DIGS and/or CIDI in the SMC, MINI and/or CIDI in the SNUBH), the items related to SA were comparable, and the rate of SA did not differ between the two institutions (Table 1). Lastly, the observation period for lifetime SA could not be fixed, and the presence of false-negative cases in the non-attempter group could not be ruled out.

## Conclusion

The present study provided additional evidence that the polygenic effects for specific traits contributed to the risk of lifetime SA in patients with BD. The PRS for SA calculated using the EUR data of BD patients showed a significant association with lifetime SAs in Korean patients with BD. In addition, the PRS for OCD seemed to affect repeated attempts. Both findings were more prominent in the BD-II subgroup, which showed a higher rate of SAs than that in the BD-I subgroup. Future genomic studies based on larger, ethnically diverse samples with more refined phenotypes are needed to identify the genetic architecture of suicide attempt.

## Supplementary Information

**Additional file 1: Table S1.** Items on lifetime suicide attempt or number of attempts in assessment tools used in the study. **Table S2.** Number of patients assessed using each tool. **Table S3.** Details of GWAS summary statistics used in the study. **Table S4.** Comparison of clinical characteristics and suicide attempts between bipolar I disorder and bipolar II disorder. **Figure S1.** No definite population structure identified by multidimensional scaling. **Figure S2.** Distribution of the number of attempts in the study patients (total N = 378). **Figure S3.** Apparent validation of the suicide attempt model utilizing five polygenic risk scores. **Figure S4.** Relationship between polygenic risk for suicide attempt and lifetime suicide attempt or number of attempts. **Figure S5.** Association of polygenic risk scores for obsessive–compulsive disorder with comorbid obsessive–compulsive disorder (N = 39 of 284 with available data) vs. no comorbid obsessive–compulsive disorder (N = 245 of 284 with available data). **Figure S6.** Association between polygenic risk scores for suicide attempt in patients with bipolar disorder, schizophrenia, or major depressive disorder and lifetime suicide attempt.

## Data Availability

Not applicable.

## Abbreviations

SA: Suicide attempt
BD: Bipolar disorder
SMR: Standardized mortality ratio
SI: Suicidal ideation
SNP: Single nucleotide polymorphism
MDD: Major depressive disorder
SCZ: Schizophrenia
ADHD: Attention-deficit/hyperactivity disorder
GWAS: Genome-wide association study
EUR: European ancestry
PRS: Polygenic risk score
PGC: Psychiatric Genomic Consortium
OCD: Obsessive–compulsive disorder
BD-I: Bipolar I disorder
BD-II: Bipolar II disorder
SMC: Samsung Medical Center
SNUBH: Seoul National University Bundang Hospital
DIGS: Diagnostic Interview for Genetic Studies
MINI: Mini-International Neuropsychiatric Interview
CIDI: Composite International Diagnostic Interview
QC: Quality control
MAF: Minor allele frequency
IOCDF-GC: International Obsessive Compulsive Disorder Foundation Genetics Collaborative
OCGAS: OCD Collaborative Genetics Association Studies
SD: Standard deviation
DV: Dependent variable
IV: Independent variable
PRS-SA: PRS for SA
OR: Odds ratio
PRS-BD: PRS for BD
PRS-SCZ: PRS for SCZ
PRS-MDD: PRS for MDD
PRS-OCD: PRS for OCD

## References

[CR1] Aaltonen K, Naatanen P, Heikkinen M, Koivisto M, Baryshnikov I, Karpov B (2016). Differences and similarities of risk factors for suicidal ideation and attempts among patients with depressive or bipolar disorders. J Affect Disord.

[CR2] Allardyce J, Leonenko G, Hamshere M, Pardinas AF, Forty L, Knott S (2018). Association between schizophrenia-related polygenic liability and the occurrence and level of mood-incongruent psychotic symptoms in bipolar disorder. JAMA Psychiatry.

[CR3] Amerio A (2019). Suicide risk in comorbid bipolar disorder and obsessive–compulsive disorder: a systematic review. Indian J Psychol Med.

[CR4] Arici C, Cremaschi L, Dobrea C, Vismara M, Grancini B, Benatti B (2018). Differentiating multiple vs single lifetime suicide attempters with bipolar disorders: a retrospective study. Compr Psychiatry.

[CR5] Baek JH, Ha K, Kim Y, Cho YA, Yang SY, Choi Y (2019). Psychopathologic structure of bipolar disorders: exploring dimensional phenotypes, their relationships, and their associations with bipolar I and II disorders. Psychol Med.

[CR6] Ballard ED, Cui L, Vandeleur C, Castelao E, Zarate CA, Preisig M (2019). Familial aggregation and coaggregation of suicide attempts and comorbid mental disorders in adults. JAMA Psychiatry.

[CR7] Bey K, Weinhold L, Grutzmann R, Heinzel S, Kaufmann C, Klawohn J (2020). The polygenic risk for obsessive–compulsive disorder is associated with the personality trait harm avoidance. Acta Psychiatr Scand.

[CR8] Bipolar Disoder and Schizophrenia Working Group of the Psychiatric Genomics Consortium (2018). Genomic dissection of bipolar disorder and schizophrenia, including 28 subphenotypes. Cell.

[CR9] Bondy B, Buettner A, Zill P (2006). Genetics of suicide. Mol Psychiatry.

[CR10] Bredemeier K, Miller IW (2015). Executive function and suicidality: a systematic qualitative review. Clin Psychol Rev.

[CR11] Brent DA, Mann JJ (2005). Family genetic studies, suicide, and suicidal behavior. Am J Med Genet C Semin Med Genet.

[CR12] Cavedini P, Zorzi C, Piccinni M, Cavallini MC, Bellodi L (2010). Executive dysfunctions in obsessive–compulsive patients and unaffected relatives: searching for a new intermediate phenotype. Biol Psychiatry.

[CR13] Chamberlain SR, Fineberg NA, Menzies LA, Blackwell AD, Bullmore ET, Robbins TW (2007). Impaired cognitive flexibility and motor inhibition in unaffected first-degree relatives of patients with obsessive–compulsive disorder. Am J Psychiatry.

[CR14] Chen YW, Dilsaver SC (1996). Lifetime rates of suicide attempts among subjects with bipolar and unipolar disorders relative to subjects with other Axis I disorders. Biol Psychiatry.

[CR15] Cho M, Hahm BJ, Suh DW, Hong JP, Bae JN, Kim J-K (2002). Development of a Korean version of the composite international diagnostic interview (K-CIDI). J Korean Neuropsychiatr Assoc.

[CR16] Choi SW, O'Reilly PF (2019). PRSice-2: polygenic risk score software for biobank-scale data. Gigascience.

[CR17] Coombes BJ, Markota M, Mann JJ, Colby C, Stahl E, Talati A (2020). Dissecting clinical heterogeneity of bipolar disorder using multiple polygenic risk scores. Transl Psychiatry.

[CR18] Cross-Disorder Group of the Psychiatric Genomics Consortium (2019). Genomic relationships, novel loci, and pleiotropic mechanisms across eight psychiatric disorders. Cell.

[CR19] Di Salvo G, Pessina E, Aragno E, Martini A, Albert U, Maina G (2020). Impact of comorbid obsessive–compulsive disorder on suicidality in patients with bipolar disorder. Psychiatry Res.

[CR20] Duncan L, Shen H, Gelaye B, Meijsen J, Ressler K, Feldman M (2019). Analysis of polygenic risk score usage and performance in diverse human populations. Nat Commun.

[CR21] Engström C, Brändström S, Sigvardsson S, Cloninger CR, Nylander PO (2004). Bipolar disorder. III: harm avoidance a risk factor for suicide attempts. Bipolar Disord.

[CR22] Erlangsen A, Appadurai V, Wang Y, Turecki G, Mors O, Werge T (2020). Genetics of suicide attempts in individuals with and without mental disorders: a population-based genome-wide association study. Mol Psychiatry.

[CR23] Fedyszyn IE, Erlangsen A, Hjorthøj C, Madsen T, Nordentoft M (2016). Repeated suicide attempts and suicide among individuals with a first emergency department contact for attempted suicide: a prospective, nationwide, Danish register-based study. J Clin Psychiatry.

[CR24] Fullerton JM, Nurnberger JI. Polygenic risk scores in psychiatry: will they be useful for clinicians? F1000Research. 2019;8:F1000 Faculty Rev-1293.

[CR25] Galfalvy H, Huang YY, Oquendo MA, Currier D, Mann JJ (2009). Increased risk of suicide attempt in mood disorders and TPH1 genotype. J Affect Disord.

[CR26] Glenn CR, Kleiman EM, Cha CB, Deming CA, Franklin JC, Nock MK (2018). Understanding suicide risk within the research domain criteria (RDoC) framework: a meta-analytic review. Depress Anxiety.

[CR27] Gonda X, Pompili M, Serafini G, Montebovi F, Campi S, Dome P (2012). Suicidal behavior in bipolar disorder: epidemiology, characteristics and major risk factors. J Affect Disord.

[CR28] Gordovez FJA, McMahon FJ (2020). The genetics of bipolar disorder. Mol Psychiatry.

[CR29] Gupta G, Deval R, Mishra A, Upadhyay S, Singh PK, Rao VR (2020). Re-testing reported significant SNPs related to suicide in a historical high-risk isolated population from north east India. Hereditas.

[CR30] Harfmann EJ, Rhyner KT, Ingram RE (2019). Cognitive inhibition and attentional biases in the affective go/no-go performance of depressed, suicidal populations. J Affect Disord.

[CR31] Howard DM, Adams MJ, Clarke TK, Hafferty JD, Gibson J, Shirali M (2019). Genome-wide meta-analysis of depression identifies 102 independent variants and highlights the importance of the prefrontal brain regions. Nat Neurosci.

[CR32] Howie B, Fuchsberger C, Stephens M, Marchini J, Abecasis GR (2012). Fast and accurate genotype imputation in genome-wide association studies through pre-phasing. Nat Genet.

[CR33] Icick R, Melle I, Etain B, Ringen PA, Aminoff SR, Leboyer M (2019). Tobacco smoking and other substance use disorders associated with recurrent suicide attempts in bipolar disorder. J Affect Disord.

[CR34] Ikeda M, Saito T, Kanazawa T, Iwata N (2021). Polygenic risk score as clinical utility in psychiatry: a clinical viewpoint. J Hum Genet.

[CR35] International Obsessive Compulsive Disorder Foundation Genetics Collaborative (IOCDF-GC) and OCD Collaborative Genetics Association Studies (OCGAS) (2018). Revealing the complex genetic architecture of obsessive–compulsive disorder using meta-analysis. Mol Psychiatry.

[CR36] Jeon HJ, Lee JY, Lee YM, Hong JP, Won SH, Cho SJ (2010). Lifetime prevalence and correlates of suicidal ideation, plan, and single and multiple attempts in a Korean nationwide study. J Nerv Ment Dis.

[CR37] Joo EJ, Joo YH, Hong JP, Hwang S, Maeng SJ, Han JH (2004). Korean version of the diagnostic interview for genetic studies: validity and reliability. Compr Psychiatry.

[CR38] Lam M, Chen CY, Li Z, Martin AR, Bryois J, Ma X (2019). Comparative genetic architectures of schizophrenia in East Asian and European populations. Nat Genet.

[CR39] Loh PR, Danecek P, Palamara PF, Fuchsberger C, Reshef YA, Finucane HK (2016). Reference-based phasing using the Haplotype Reference Consortium panel. Nat Genet.

[CR40] Lopes FL, Zhu K, Purves KL, Song C, Ahn K, Hou L (2020). Polygenic risk for anxiety influences anxiety comorbidity and suicidal behavior in bipolar disorder. Transl Psychiatry.

[CR41] Malhi GS, Outhred T, Das P, Morris G, Hamilton A, Mannie Z (2018). Modeling suicide in bipolar disorders. Bipolar Disord.

[CR42] Manichaikul A, Mychaleckyj JC, Rich SS, Daly K, Sale M, Chen WM (2010). Robust relationship inference in genome-wide association studies. Bioinformatics.

[CR43] Mann JJ, Apter A, Bertolote J, Beautrais A, Currier D, Haas A (2005). Suicide prevention strategies: a systematic review. JAMA.

[CR44] Martin AR, Kanai M, Kamatani Y, Okada Y, Neale BM, Daly MJ (2019). Clinical use of current polygenic risk scores may exacerbate health disparities. Nat Genet.

[CR45] McCarthy S, Das S, Kretzschmar W, Delaneau O, Wood AR, Teumer A (2016). A reference panel of 64,976 haplotypes for genotype imputation. Nat Genet.

[CR46] Menzies L, Achard S, Chamberlain SR, Fineberg N, Chen CH, del Campo N (2007). Neurocognitive endophenotypes of obsessive–compulsive disorder. Brain.

[CR47] Moon S, Kim YJ, Han S, Hwang MY, Shin DM, Park MY (2019). The Korea biobank array: design and identification of coding variants associated with blood biochemical traits. Sci Rep.

[CR48] Mullins N, Perroud N, Uher R, Butler AW, Cohen-Woods S, Rivera M (2014). Genetic relationships between suicide attempts, suicidal ideation and major psychiatric disorders: a genome-wide association and polygenic scoring study. Am J Med Genet B Neuropsychiatr Genet.

[CR49] Mullins N, Bigdeli TB, Børglum AD, Coleman JRI, Demontis D, Mehta D (2019). GWAS of suicide attempt in psychiatric disorders and association with major depression polygenic risk scores. Am J Psychiatry.

[CR50] Novick DM, Swartz HA, Frank E (2010). Suicide attempts in bipolar I and bipolar II disorder: a review and meta-analysis of the evidence. Bipolar Disord.

[CR51] Otsuka I, Akiyama M, Shirakawa O, Okazaki S, Momozawa Y, Kamatani Y (2019). Genome-wide association studies identify polygenic effects for completed suicide in the Japanese population. Neuropsychopharmacology.

[CR52] Ozcan H, Ozer S, Yagcioglu S (2016). Neuropsychological, electrophysiological and neurological impairments in patients with obsessive compulsive disorder, their healthy siblings and healthy controls: identifying potential endophenotype(s). Psychiatry Res.

[CR53] Papadopoulou A, Efstathiou V, Christodoulou C, Gournellis R, Papageorgiou C, Douzenis A (2020). Psychiatric diagnosis, gender, aggression, and mode of attempt in patients with single versus repeated suicide attempts. Psychiatry Res.

[CR54] Pellegrini L, Maietti E, Rucci P, Casadei G, Maina G, Fineberg NA (2020). Suicide attempts and suicidal ideation in patients with obsessive–compulsive disorder: a systematic review and meta-analysis. J Affect Disord.

[CR55] Plans L, Barrot C, Nieto E, Rios J, Schulze TG, Papiol S (2019). Association between completed suicide and bipolar disorder: a systematic review of the literature. J Affect Disord.

[CR56] Pompili M, Gonda X, Serafini G, Innamorati M, Sher L, Amore M (2013). Epidemiology of suicide in bipolar disorders: a systematic review of the literature. Bipolar Disord.

[CR57] Purcell S, Neale B, Todd-Brown K, Thomas L, Ferreira MA, Bender D (2007). PLINK: a tool set for whole-genome association and population-based linkage analyses. Am J Hum Genet.

[CR58] R Development Core Team (2010). R: a language and environment for statistical computing.

[CR59] Rajender G, Bhatia MS, Kanwal K, Malhotra S, Singh TB, Chaudhary D (2011). Study of neurocognitive endophenotypes in drug-naïve obsessive–compulsive disorder patients, their first-degree relatives and healthy controls. Acta Psychiatr Scand.

[CR60] Rao S, Shi M, Han X, Lam MHB, Chien WT, Zhou K (2020). Genome-wide copy number variation-, validation- and screening study implicates a new copy number polymorphism associated with suicide attempts in major depressive disorder. Gene.

[CR61] Rawat S, Rajkumari S, Joshi PC, Khan MA, Saraswathy KN (2019). Risk factors for suicide attempt: a population-based-genetic study from Telangana, India. Curr Psychol.

[CR62] Roy A, Segal NL (2001). Suicidal behavior in twins: a replication. J Affect Disord.

[CR63] Ruderfer DM, Fanous AH, Ripke S, McQuillin A, Amdur RL, Schizophrenia Working Group of the Psychiatric Genomics C (2014). Polygenic dissection of diagnosis and clinical dimensions of bipolar disorder and schizophrenia. Mol Psychiatry.

[CR64] Ruderfer DM, Walsh CG, Aguirre MW, Tanigawa Y, Ribeiro JD, Franklin JC (2020). Significant shared heritability underlies suicide attempt and clinically predicted probability of attempting suicide. Mol Psychiatry.

[CR65] Schaffer A, Isometsä ET, Tondo L, Moreno DH, Turecki G, Reis C (2015). International society for bipolar disorders task force on suicide: meta-analyses and meta-regression of correlates of suicide attempts and suicide deaths in bipolar disorder. Bipolar Disord.

[CR66] Sidorchuk A, Kuja-Halkola R, Runeson B, Lichtenstein P, Larsson H, Ruck C (2021). Genetic and environmental sources of familial coaggregation of obsessive–compulsive disorder and suicidal behavior: a population-based birth cohort and family study. Mol Psychiatry.

[CR67] Stahl EA, Breen G, Forstner AJ, McQuillin A, Ripke S, Trubetskoy V (2019). Genome-wide association study identifies 30 loci associated with bipolar disorder. Nat Genet.

[CR68] Statham DJ, Heath AC, Madden PA, Bucholz KK, Bierut L, Dinwiddie SH (1998). Suicidal behaviour: an epidemiological and genetic study. Psychol Med.

[CR69] Tondo L, Lepri B, Baldessarini RJ (2007). Suicidal risks among 2826 Sardinian major affective disorder patients. Acta Psychiatr Scand.

[CR70] Tondo L, Pompili M, Forte A, Baldessarini RJ (2016). Suicide attempts in bipolar disorders: comprehensive review of 101 reports. Acta Psychiatr Scand.

[CR71] Too LS, Spittal MJ, Bugeja L, Reifels L, Butterworth P, Pirkis J (2019). The association between mental disorders and suicide: a systematic review and meta-analysis of record linkage studies. J Affect Disord.

[CR72] Vieta E, Benabarre A, Colom F, Gasto C, Nieto E, Otero A (1997). Suicidal behavior in bipolar I and bipolar II disorder. J Nerv Ment Dis.

[CR73] Voracek M, Loibl LM (2007). Genetics of suicide: a systematic review of twin studies. Wien Klin Wochenschr.

[CR74] Yoo SW, Kim Y, Noh JS (2006). Validity of Korean version of the mini-international neuropsychiatric interview. Anxiety Mood.

[CR75] Zhang L, Dong Y, Ji Y, Zhu C, Yu F, Ma H (2015). Dissociation of decision making under ambiguity and decision making under risk: a neurocognitive endophenotype candidate for obsessive–compulsive disorder. Prog Neuropsychopharmacol Biol Psychiatry.

