# Supplementary material for: Dissecting the genetic architecture of suicide attempt and repeated attempts in Korean patients with bipolar disorder using polygenic risk scores
Source: Int J Bipolar Disord. 2022 Feb 3;10:3. doi: 10.1186/s40345-022-00251-x (PMC8811109; doi:10.1186/s40345-022-00251-x)
Supplement: Supplementary file 1 — Additional file 1: Table S1. Items on lifetime suicide attempt or number of attempts in assessment tools used in the study. Table S2. Number of patients assessed using each tool. Table S3. Details of GWAS summary statistics used in the study. Table S4. Comparison of clinical characteristics and suicide attempts between bipolar I disorder and bipolar II disorder. Figure S1. No definite population structure identified by multidimensional scaling. Figure S2. Distribution of the number of attempts in the study patients (total N = 378). Figure S3. Apparent validation of the suicide attempt model utilizing five polygenic risk scores. Figure S4. Relationship between polygenic risk for suicide attempt and lifetime suicide attempt or number of attempts. Figure S5. Association of polygenic risk scores for obsessive–compulsive disorder with comorbid obsessive–compulsive disorder (N = 39 of 284 with available data) vs. no comorbid obsessive–compulsive disorder (N = 245 of 284 with available data). Figure S6. Association between polygenic risk scores for suicide attempt in patients with bipolar disorder, schizophrenia, or major depressive disorder and lifetime suicide attempt. [file 40345_2022_251_MOESM1_ESM.docx]

Additional file 1

**Contents**

**Additional Tables2**

Table S1. Items on lifetime suicide attempt or number of attempts in assessment tools used in the study2

Table S2. Number of patients assessed using each tool3

Table S3. Details of GWAS summary statistics used in the study4

Table S4. Comparison of clinical characteristics and suicide attempts between bipolar I disorder and bipolar II disorder5

**Additional Figures6**

Figure S1. No definite population structure identified by multidimensional scaling 6

Figure S2. Distribution of the number of attempts in the study patients (total N = 378)7

Figure S3. Apparent validation of the suicide attempt model utilizing five polygenic risk scores8

Figure S4. Relationship between polygenic risk for suicide attempt and lifetime suicide attempt or number of attempts.9

Figure S5. Association of polygenic risk scores for obsessive-compulsive disorder with comorbid obsessive-compulsive disorder (N = 39 of 284 with available data) vs no comorbid obsessive-compulsive disorder (N = 245 of 284 with available data) ^a^.10

Figure S6. Association between polygenic risk scores for suicide attempt in patients with bipolar disorder, schizophrenia, or major depressive disorder and lifetime suicide attempt11

**References12**

**Table S1 Items on lifetime suicide attempt or number of attempts in assessment tools used in the study**

| **Tool** | **Item** | **Question** |
| --- | --- | --- |
| DIGS | Lifetime suicide attempt | Have you ever tried to kill yourself? |
|  | Number of attempts | How many times have you tried to kill yourself? |
| MINI | Lifetime suicide attempt | Did you ever make a suicide attempt? |
|  | Number of attempts | Number of suicide attempts when in depressive episodes |
|  |  | Number of suicide attempts when not in depressive episodes |
| CIDI | Lifetime suicide attempt | Did experience C ever happen to you? |
|  |  | Experience C is “You attempted suicide.” |
|  | Number of attempts | How many times did experience C happen to you in your lifetime? |

*DIGS* Diagnostic Interview for Genetic Studies, *MINI* Mini International Neuropsychiatric Interview, *CIDI* Composite International Diagnostic Interview

**Table S2 Number of patients assessed using each tool**

| **Center** | **Assessment tool** | **Number of patients** | |
| --- | --- | --- | --- |
|  |  | **Suicide attempt** | **Number of attempts** |
| Samsung Medical Center | DIGS only | 95 | 95 |
|  | CIDI only | 6 | 6 |
|  | DIGS and CIDI | 111 | 111 |
|  | Subtotal | 212 | 212 |
| Seoul National University Bundang Hospital | MINI only | 6 | 10 |
|  | CIDI only | 89 | 88 |
|  | MINI and CIDI | 76 | 68 |
|  | Subtotal | 171 | 166 |
| Total | | 383 | 378 |

*DIGS* Diagnostic Interview for Genetic Studies, *MINI* Mini International Neuropsychiatric Interview, *CIDI* Composite International Diagnostic Interview

**Table S3 Details of GWAS summary statistics used in the study**

| **Phenotype** | **Study authors, year, reference** | **Case** | **Control** | **Population** | **SNP heritability^a^** |
| --- | --- | --- | --- | --- | --- |
| Suicide attempt^b^ | Mullins et al., (2019) | 3264 | 5500 | European | 0.02 ± 0.03 |
| Bipolar disorder | Stahl et al., (2019) | 29764 | 169118 | European | 0.20 ± 0.03 |
| Schizophrenia | Lam et al., (2019) | European 33,640 + East Asian 22,778 | European 43,456 + East Asian 35,362 | European + East Asian | European : 0.24 ± 0.02 East Asian : 0.23 ± 0.03 |
| Major depressive disorder^c^ | Howard et al., (2019) | 170,756 | 329,443 | European | 0.089 ± 0.003 |
| Obsessive-compulsive disorder | IOCDF-GC and OCGAS, (2018) | 2688 | 7037 | European | 0.28 ± 0.04 |

^a^Linkage disequilibrium score regression method was used except for the study of Mullins et al. where the genome-wide complex trait analysis (GCTA) method was used.

^b^Results from the samples including only bipolar disorder patients were used.

^c^Data from 23andMe was not included due to restrictions in the data transfer agreement.

|  | **Bipolar I disorder (n = 225)** | | **Bipolar II disorder (n = 158)** | |  |
| --- | --- | --- | --- | --- | --- |
|  | **Mean** | **SD** | **Mean** | **SD** | ***p*^a^** |
| Age | 33.8 | 10.2 | 36.3 | 11.6 | 0.026 |
| Lifetime number of suicide attempts | 0.4 | 0.9 | 1.0 | 2.0 | <0.001 |
|  | **N** | **%** | **N** | **%** | *p***^b^** |
| Sex, female | 136 | 60.4 | 113 | 71.5 | 0.033 |
| Comorbid obsessive-compulsive disorder (available N = 284) | 25 | 13.7 | 14 | 13.9 | 1.000 |
| Lifetime suicide attempt | 56 | 24.9 | 65 | 41.1 | 0.001 |
| ^a^T-test was used ^b^Chi-squared test was used |  |  |  |  |  |

**Table S4 Comparison of clinical characteristics and suicide attempts between bipolar I disorder and bipolar II disorder**

**Figure S1.** No definite population structure was identified by multidimensional scaling^a^.


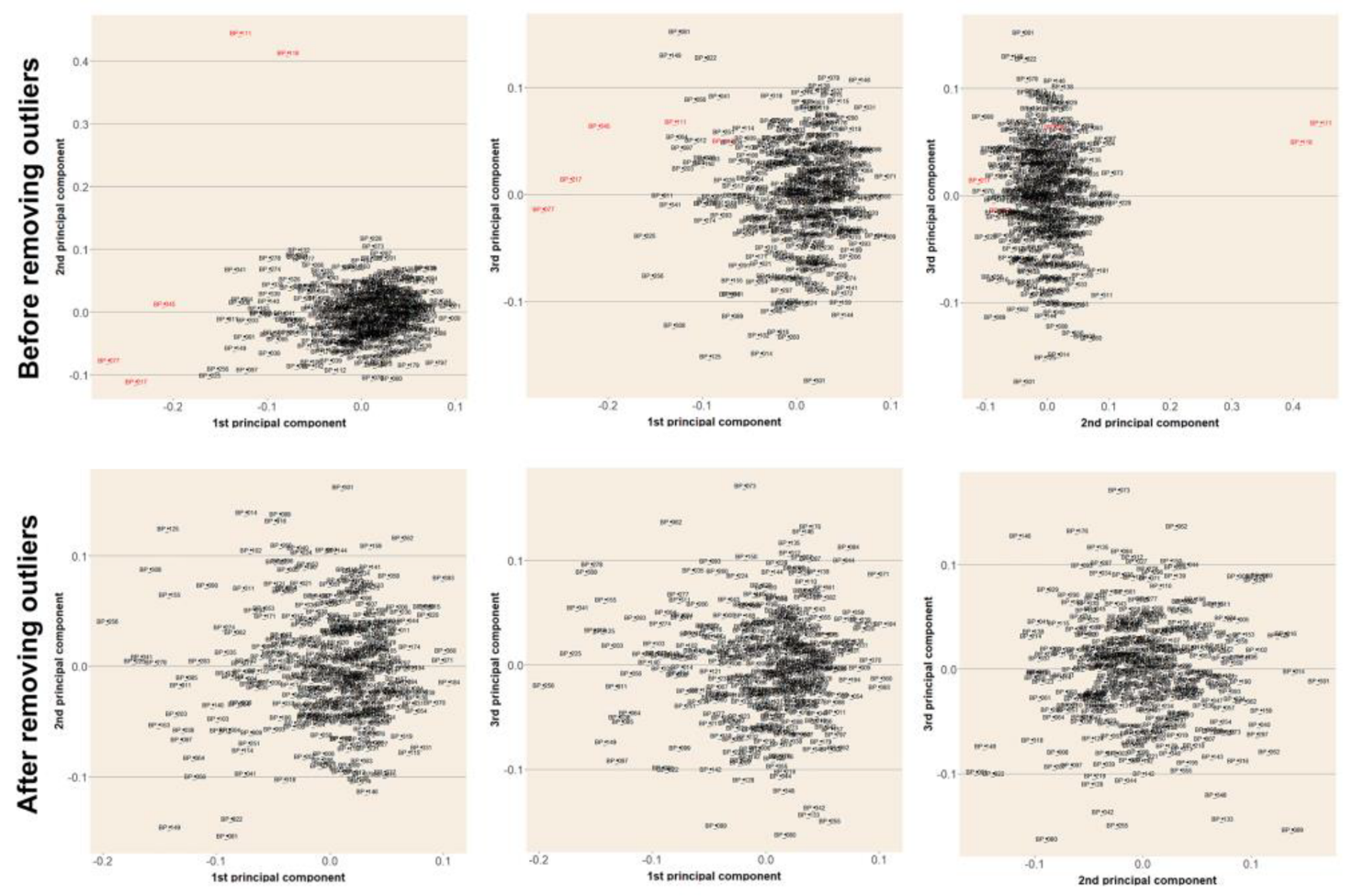


^a^The five samples with red-colored IDs were conservatively regarded as outliers and removed before recalculation of the principal components.

**Figure S2.** Distribution of the number of suicide attempts in the study patients (total N = 378).

^
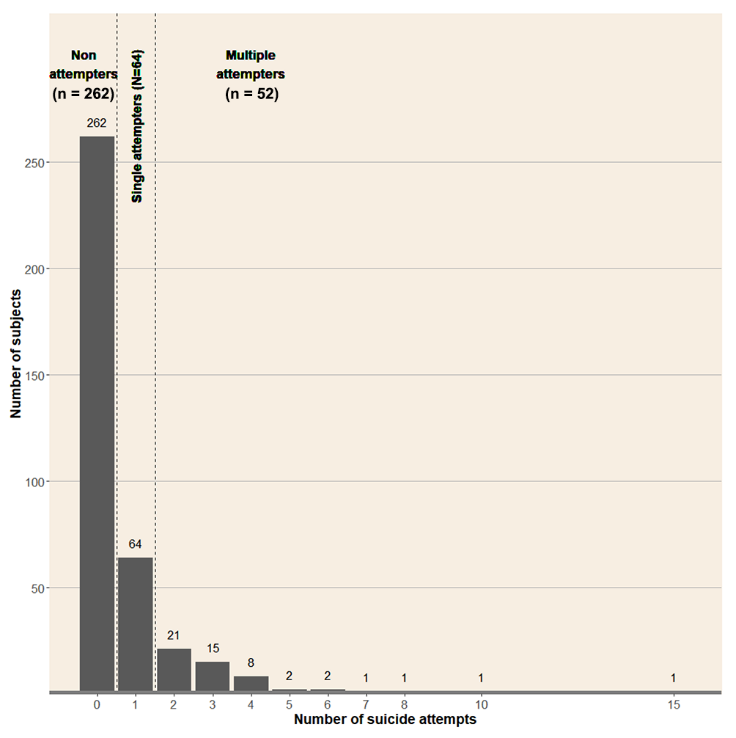
^

**Figure S3.** Apparent validation of the suicide attempt model utilizing five polygenic risk scores^a^.

^
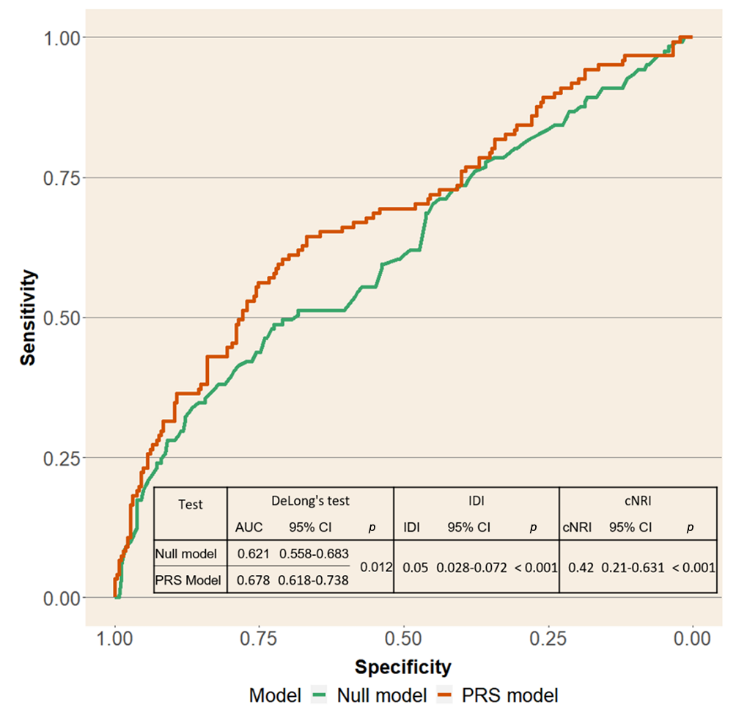
^

^a^The green curve and orange curve illustrate the receiver operating curve of the null model and the PRS model, respectively. The null model is a multivariate logistic regression model using age, sex, and diagnosis. The PRS model is a multivariate logistic regression model using age, sex, diagnosis, PRS for suicide attempt, PRS for bipolar disorder, PRS for schizophrenia, PRS for major depressive disorder, and PRS for obsessive-compulsive disorder. PRS=polygenic risk score; AUC=area under the curve; IDI=integrated discrimination index; cNRI=continuous net reclassification index

**Figure S4.** Relationship between polygenic risk for suicide attempts and lifetime suicide attempt or number of attempts^a^.


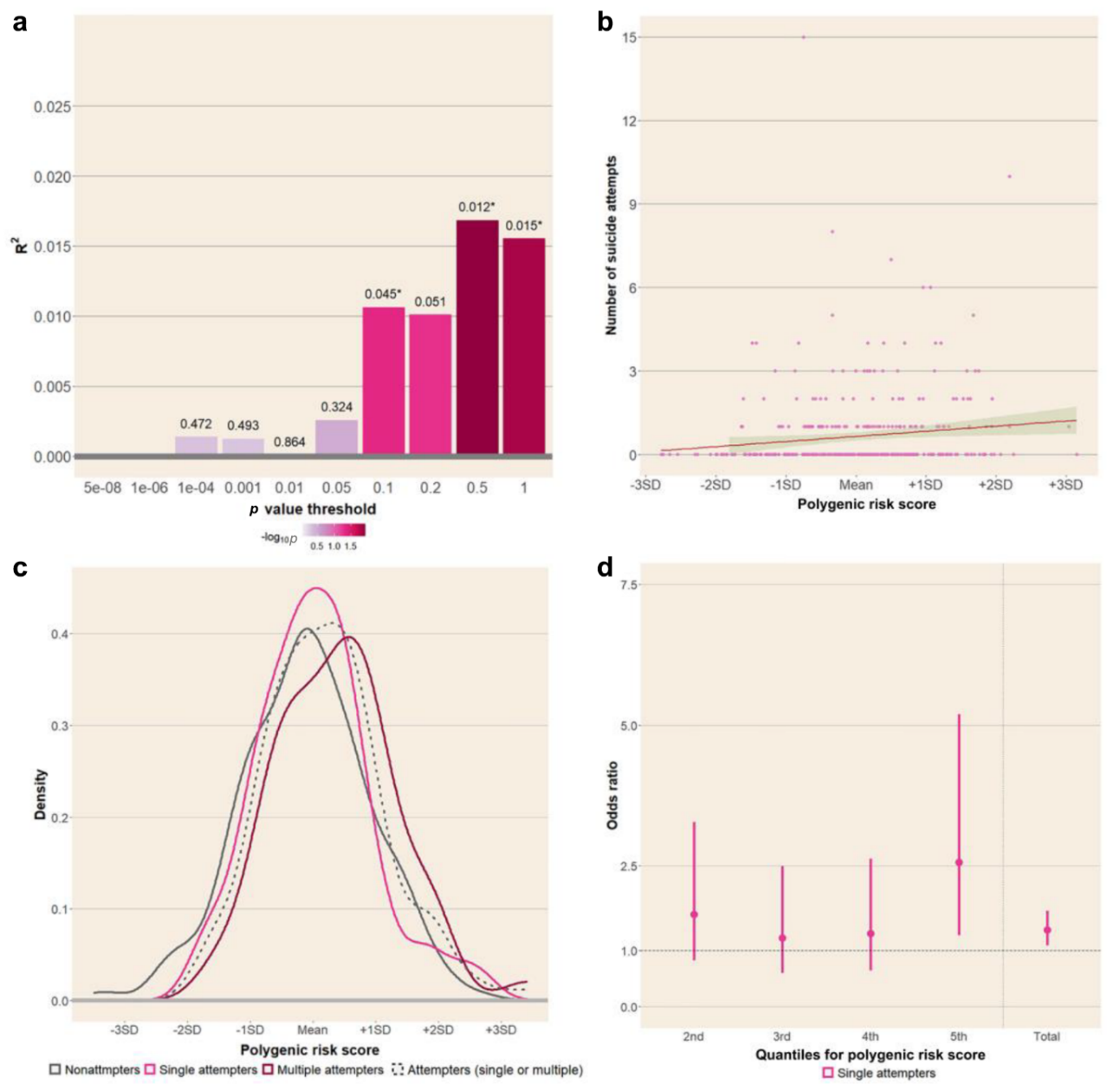


^a^(Panel A) Linear regression was performed with the PRS for SA as the independent variable and the number of attempts as the dependent variable. The x-axis illustrates the p-value thresholds used to filter the variants from the GWAS for SA. The y-axis illustrates the R^2^, which represents the proportion of the variance explained. The *P* values for the associations are shown above each bar. *Asterisk indicates nominal significance (*p* < 0.05). The model using a p value threshold of 0.5 was selected as the best-fit model. (Panel B) Scatter plot illustrates the relationship between the PRS for SA and the number of attempts. The red line and green shading illustrate the best-fit linear regression model and 95% confidence interval, respectively. The x-axis illustrates the PRS calculated using the best-fit model. Since the PRS was standardized, the x-axis ticks were marked with the mean and standard deviation. (Panel C) Density plot illustrates the distribution of the PRS for SA across non-attempters (N = 262), single attempters (N = 64), multiple attempters (N = 52), and lifetime attempters (single or multiple) (N = 121). The PRS for SA was significantly different between non-attempters vs attempters, but not between single attempters vs multiple attempters. (Panel D) Quantile plot illustrates the pattern of the ORs for attempters vs non-attempters across the quantiles of the PRS for SA. The x-axis illustrates the last four quantiles of all five quantiles of the PRS for SA. The y-axis illustrates the OR for attempters vs single attempters in each quantile compared to the first quantile. The OR from the total sample was also shown after the dotted vertical line. The points and lines illustrate ORs and 95 % confidence intervals, respectively. PRS=polygenic risk score; SA=suicide attempt; GWAS=genome-wide association study; OR=odds ratio

**Figure S5.** Association of polygenic risk scores for obsessive-compulsive disorder with comorbid obsessive-compulsive disorder (N = 39 of 284 with available data) vs no comorbid obsessive-compulsive disorder (N = 245 of 284 with available data)^a^.

^
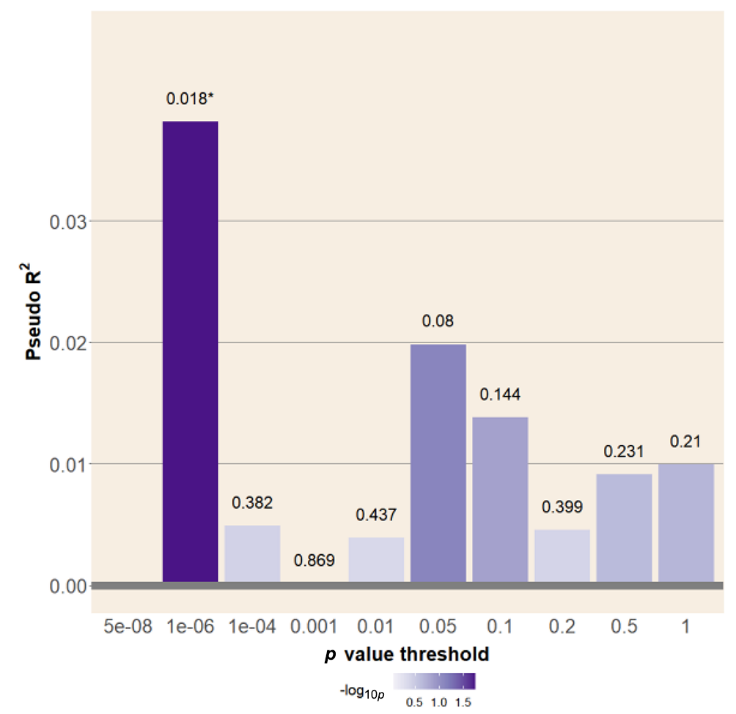
^

^a^Logistic regression was performed with the polygenic risk score for obsessive-compulsive disorder as the independent variable and with comorbid obsessive-compulsive disorder (N = 39 of 284 with available data) as the dependent variable. The x-axis illustrates the p value thresholds used to the filter variants found in the genome-wide association study for obsessive-compulsive disorder. The y-axis illustrates Nagelkerke’s pseudo R^2^. The *P* values for the associations are shown above each bar. *Asterisk indicates nominal significance (*p* < 0.05).

**Figure S6.**  Association between polygenic risk scores for suicide attempt in patients with bipolar disorder, schizophrenia, or major depressive disorder and lifetime suicide attempt ^a^.


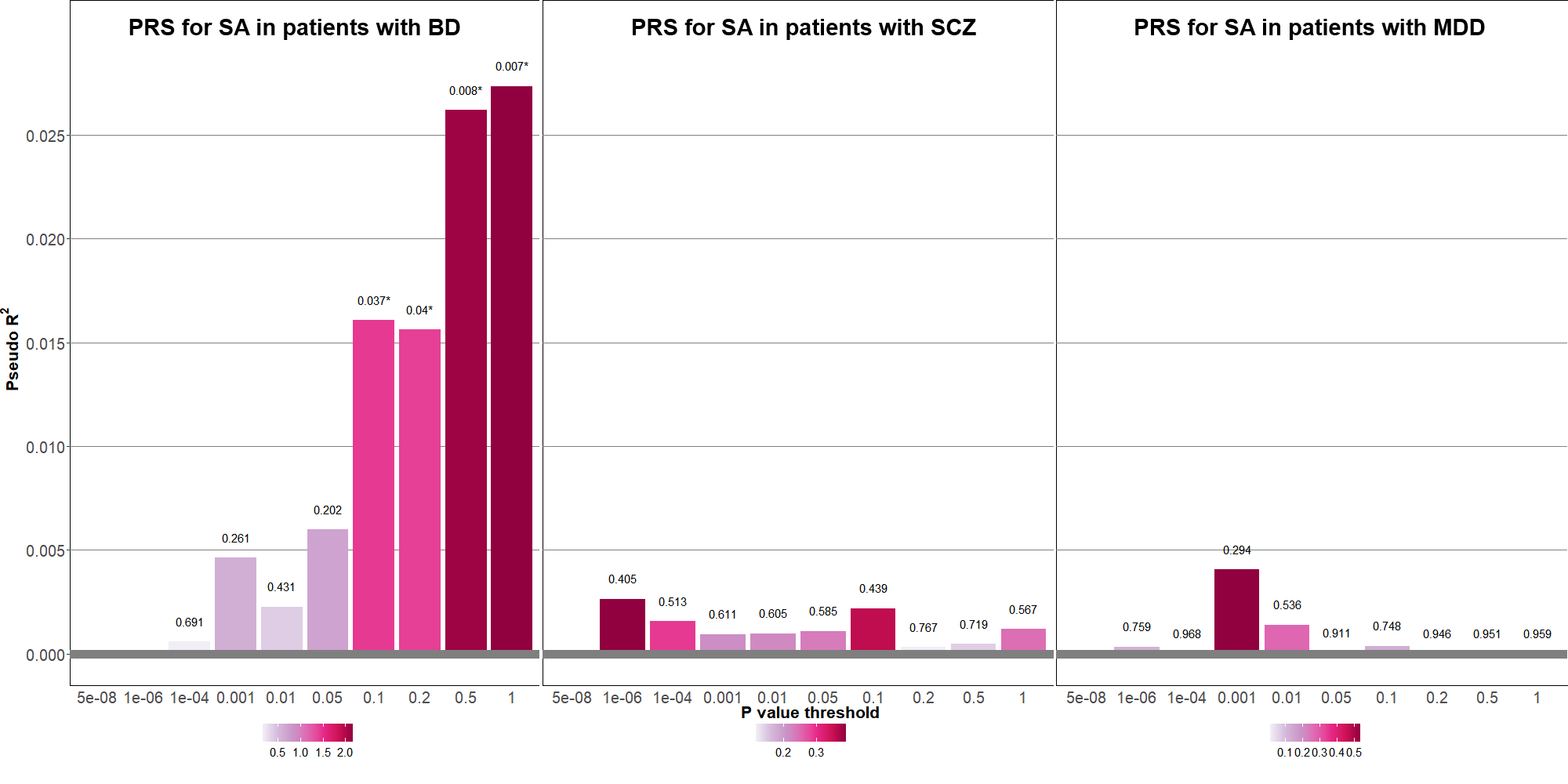


^a^ PRS for SA was calculated using reference GWAS results in patients with bipolar disorder, schizophrenia, or major depressive disorder, respectively. Logistic regression was performed with the PRS for suicide attempt as the independent variable and suicide attempters (N = 121) vs nonattempters (N = 262) as the dependent variable. The x-axis illustrates the p-value thresholds used to filter the variants from GWAS for suicidal attempt. The y-axis illustrates Nagelkerke’s pseudo R^2^. The *p* values for the associations are shown above each bar. *Asterisk indicates nominal significance (*p* < 0.05). PRS = polygenic risk score; GWAS = genome-wide association study. SA=suicide attempt, BD=bipolar disorder, SCZ=schizophrenia, MDD=major depressive disorder.

**References**

Howard DM, Adams MJ, Clarke TK, Hafferty JD, Gibson J, Shirali M, et al. Genome-wide meta-analysis of depression identifies 102 independent variants and highlights the importance of the prefrontal brain regions. Nat Neurosci. 2019;22(3):343-52.

International Obsessive Compulsive Disorder Foundation Genetics Collaborative (IOCDF-GC) and OCD Collaborative Genetics Association Studies (OCGAS). Revealing the complex genetic architecture of obsessive-compulsive disorder using meta-analysis. Mol Psychiatry. 2018;23(5):1181-8.

Lam M, Chen CY, Li Z, Martin AR, Bryois J, Ma X, et al. Comparative genetic architectures of schizophrenia in East Asian and European populations. Nat Genet. 2019;51(12):1670-8.

Mullins N, Bigdeli TB, Børglum AD, Coleman JRI, Demontis D, Mehta D, et al. GWAS of Suicide Attempt in Psychiatric Disorders and Association With Major Depression Polygenic Risk Scores. Am J Psychiatry. 2019;176(8):651-60.

Stahl EA, Breen G, Forstner AJ, McQuillin A, Ripke S, Trubetskoy V, et al. Genome-wide association study identifies 30 loci associated with bipolar disorder. Nat Genet. 2019;51(5):793-803.
